# Conventional vs. Organic Agriculture–Which One Promotes Better Yields and Microbial Resilience in Rapidly Changing Climates?

**DOI:** 10.3389/fmicb.2022.903500

**Published:** 2022-06-09

**Authors:** Hamed Azarbad

**Affiliations:** Department of Biology, Evolutionary Ecology of Plants, Philipps-University Marburg, Marburg, Germany

**Keywords:** crop, complex microbiome, yield, climate changes, agroecosystem, land-use

## Abstract

In recent years, agricultural productivity has been affected dramatically by climate-related events such as drought. On the other hand, agricultural intensification is expected to increase to satisfy the need for increased global food production. Microbes associated with soil and plants produce a range of bioactive natural products that significantly contribute to crop stress tolerance. Therefore, a better understanding of the parallel effects of agricultural management (conventional and organic croplands) and climate conditions on soil-microbe-plant interactions is crucial to maximizing the effort in engineering a plant microbiome that can better support productivity in agroecosystems. This paper provides a general overview of the major current debates on conventional and organic farming performance regarding yields, particularly under ambient and future climate conditions. With the main focus on cropland, the effect of agricultural management on soil and plant microbiomes is discussed. In addition, the advantage of incorporating microbiome-based approaches into current farming practices to ensure agricultural productivity with less adverse environmental impacts is highlighted. To enhance crop production under organic farming without massive land-use changes and expansion of farmland, the microbial-based approach can be used to ensure higher productivity, particularly under a rapidly changing climate.

## Introduction

The agricultural area covers around 38% of the global land surface, where one-third of this is used as cropland, and the rest, including meadows and pastures serves as grazing land (FAO, 2020). Agricultural intensification is expected to increase due to the projected increased global food production by up to 70% by 2050 (FAO, 2009). Therefore, high pressure on farming systems will continue to feed a growing human population. Massive land-use changes and expansion of farmland lead to several negative consequences at the global level, such as land degradation, habitat and biodiversity losses, and enhancing greenhouse gas emissions (Foley, 2005; Reganold and Wachter, 2016; United Nations Environment Programme, 2016; Powers and Jetz, 2019). In addition, climate change-related stressors are likely to amplify the negative impacts of such human activities on various ecosystem functions (IPBES, 2019). Increased frequency and severity of extreme weather events are among the most substantial effects of climate change (IPCC, 2014). For instance, in Germany, drought has increased substantially in recent years and reduced crop yields (Eckstein et al., 2020). Therefore, it is important to understand better the impact of multiple anthropogenic and global climate change stressors on agroecosystems since the cumulative effects and interaction between such disturbances can substantially decrease crop production.

**FIGURE 1:**
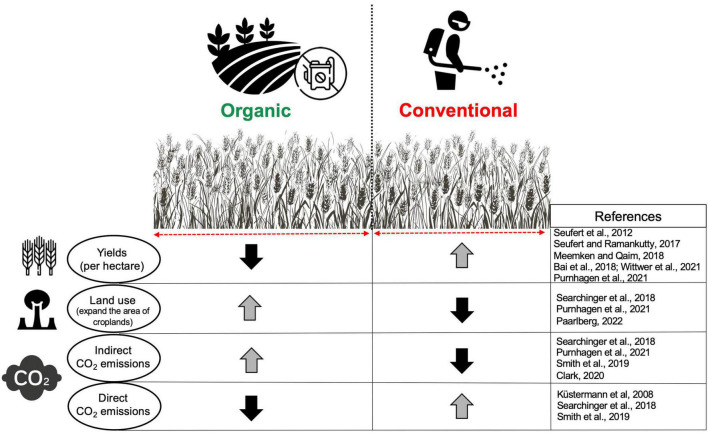
The comparisons between organic and conventional agriculture in terms of yield and environmental effects. The direction of the arrows indicates an increase or decrease in the considered factors.

Conventional farming (CF) includes a large amount of chemical fertilizer and pesticide use to increase the yields per hectare. Chemical and synthetic fertilizers and pesticides are not applied in the organic farming (OF) system to reduce their adverse environmental impacts. Instead, plant residues or livestock manure are used to enhance soil fertility (Lori et al., 2017). Based on the Farm to Fork (F2F) Strategy under the “European Green Deal” which was established in December 2019 by the European Commission, agricultural land farmed organically would need to increase from the current 9% of the total utilized agricultural land to 25% by 2030. It has been shown recently that compared with CF, OF reduces soil erosion and aquatic ecotoxicity potential (Wittwer et al., 2021). In addition, OF results in more soil biodiversity and an abundance of macro and microorganisms along with higher income per hectare, but, depending on the crops, less yield (–22%) showing a trade-off between environmental protection and agricultural productivity (Wittwer et al., 2021). Supporting this view, it has been reported that under experimental and field conditions, yields (per hectare) from OF may reduce up to 20–25% and 50% (respectively) in comparison with CF (Seufert and Ramankutty, 2017; Meemken and Qaim, 2018). Therefore, yield production is one of the main limitations of OF, which would mean that more land should be farmed organically to produce the same amount of yield as CF to satisfy food demands (Figure 1). Such land-use changes may result in more deforestation and other ecosystem loss, leading to indirectly contributing to carbon dioxide emissions due to greater soil organic carbon release into the atmosphere (Paarlberg, 2022). Since organic agriculture will be an essential part of the farming system in Europe and worldwide, it is crucial to better understand the long-term benefits and limitations of such a system from different perspectives, particularly on soil processes and biodiversity under changing climate.

When the effect of agricultural management on yields under ambient and future climate conditions is compared, the story becomes even more complicated. Agroecosystems are under severe threat from crop pathogens and pests, which can be more problematic under OF since synthetic pesticides and biotechnology innovations are not applicable. According to models and predictions, such threats will increase by 2050, mainly due to the rapidly changing climate, particularly for crops in tropical countries (Bebber et al., 2014). Although it has been demonstrated that compared to different land management practices, the existing conventionally managed croplands promote crop yields (given a local climate condition across the world) without a need for land use changes and expansion of cropland area (Licker et al., 2010). However, such agricultural practice demands more chemical, fertilizer, and water inputs to keep the yield at a high level, thus negatively impacting the ecosystem (Licker et al., 2010). On the other hand, by conducting a 5-year field trial, Lotter et al. (2003) provided evidence that OF resulted in significantly higher yields than CF under severe drought events, thus fostering sustainable food production. They conclude that such effects are mainly due to the higher water capacity of soil under OF, helping the plant to better cope with drought stress. Given these contradictory results and based on the F2F strategy that aims to increase OF, the question then becomes: how does OF withstand severe threats to global agriculture to secure food production, considering that food demand continues to increase? In their recent study, Purnhagen et al. (2021) discussed the advantage of incorporating novel plant breeding technologies (such as CRISPR/Cas9) with organic farming, resulting in more sustainable agriculture systems. Although plant breeding can help plants better tolerate suboptimal conditions (Coleman-Derr and Tringe, 2014), the yield of wheat increased very slowly (approximately 1% annually) through the genetic improvement (Sayre et al., 1997), which is way far from fulfilling the increasing food demand. Therefore, novel and fast-responding approaches for enhancing agricultural productivity are needed.

Microorganisms (i.e., bacteria, fungi, and archea) associated with soil and plants produce a range of bioactive natural products that can significantly contribute to crop stress tolerance. Due to various unique characteristics, which will be discussed throughout this paper, the response of soil and plant microbes to the management practices and climate change factors needs to be considered in parallel when the aim is to maintain important biological functions (e.g., nutrients turnover in the soil) and productivity (e.g., yield) in the agroecosystem. By taking into account mainly the ecological aspects, this article examines how direct and indirect interactions between different agricultural practices and climate change may impact microbes associated with soil and crop plants. In the following sections, I summarize recent works mainly conducted on croplands to evaluate the effect of organic and conventional farming systems on soil microbiomes as the leading player in maintaining or enhancing soil quality. Then, I will discuss the possible consequences of agricultural management on soil microbial stability in the face of global change disturbances. I will explore different possible scenarios on how soil microbes under organic and conventional farming may respond to the secondary stressors, in terms of sudden or their responses over time. Next, the impact of different agricultural management on plant microbiomes will be discussed. Special attention was given, whenever possible, to those studies that examined the effect of the long-term history of conventional and organic management on the resistance and resilience of soil and plant microbiomes to one or a few stressors linked with climate change. Finally, I summarize a few microbiome-based approaches that can be incorporated into agricultural practices to enhance crop productivity.

## The Effect of Agricultural Management on the Soil Microbiomes

Soil represents the most complex and rich habitat on earth that consists of an enormous diversity of organisms (Bardgett and van der Putten, 2014). Among soil biota, microorganisms are a critical part of ecosystems due to their roles in almost all soil processes and functioning (Barrios, 2007). For example, soil microbial communities are the main drivers of nutrient cycling and decomposition of organic matter (Doran and Zeiss, 2000), inhibiting pathogens and protecting plants against stresses (Li et al., 2019). Therefore, in the context of agricultural management, the conservation of soil biota, including soil microbiome, is critical to maintaining agricultural productivity. The principle of intensive agricultural management, such as CF, is to use a high level of mineral or chemical fertilizers, regular soil tillage, and intensive pesticide and herbicide applications. In that way, although CF leads to an increase in crop production (Alaru et al., 2014), this can result in several adverse effects on soil physicochemical properties (Dubey et al., 2019), which can, in turn, impact soil microbiomes (Lupatini et al., 2017).

A large volume of research, including several reviews and meta-analysis studies, has been undertaken to evaluate the effects of different agricultural practices on soil bacterial and fungal communities. A meta-analysis by Lori et al. (2017) illustrated that OF promotes total microbial abundance and activities (e.g., dehydrogenase, protease, and urease) in comparison with CF. Using a long-term field experiment, Martínez-García et al. (2018) investigated the effect of more than 10 years of organic and conventional management on the biomass and structure of soil microbial communities (based on phospholipid fatty acids (PLFA) extraction method) and microbial catabolic response profile (based on MicroResp). Their results showed that soil that was managed organically increased bacterial and fungi (saprotrophic and arbuscular mycorrhizal) biomass, together with an increase in total microbial catabolic activity. Similarly, Goel et al. (2021), under a field trial of rice-wheat cropping system, showed that OF substantially increased the abundance of the 16S rDNA gene (assessed using qRT-PCR) and bacterial diversity (assessed using denaturant gradient gel electrophoresis-DGGE). Lupatini et al. (2017) have evaluated the long-term responses of soil bacterial communities (over 7 years since the treatment started) to organic and conventional farming systems using the 16S rRNA gene sequencing approach. They reported that OF enhanced bacterial diversity, richness, and community heterogeneity (the higher beta diversity) than the conventional system.

The Global Change Experimental Facility (GCEF) is one of the first attempts to assess the parallel effects of predicted future climate scenarios (reduced precipitation and warming) and land-use types and intensities (including conventional and organic management) on various ecosystem processes. The GCEF is part of the field research station of the Helmholtz-Centre for Environmental Research in Bad Lauchstädt, Saxony-Anhalt, Germany, established in 2014 (Schädler et al., 2019). Sünnemann et al. (2021b) reported no significant interactive impact of land-use types (conventional versus organic farming) and climate change on soil microbial activity (decomposition processes) following 2 and 3 years since the establishment of the field (Sünnemann et al., 2021b). In another study done by Sünnemann et al. (2021a) under the same experimental set-up, soil microbial respiration rates and microbial biomass (determined by PLFA profiles) remained largely unaffected by future climate conditions when soils were sampled two times in autumn for 5 years (2015–2019). However, their result indicated an increase in the abundance of arbuscular mycorrhizal fungi and fungal-to-bacterial ratios under OF than in the CF (Sünnemann et al., 2021a). Collectively, their results revealed that the beneficial effect of organic-based management needed several years to establish. However, it’s difficult to draw a general conclusion since very few such studies exist that evaluated the parallel effects of a contrasting history of agricultural management and climate conditions on soil microbiomes. Therefore, there is a need to increase efforts to understand better how management strategies affect the capacity of agricultural ecosystems to maintain their state and function under an altered climate.

## The Interaction Between Soil Management History and Microbial Stability to Environmental Stressors

Changes in soil microbiomes resulting from agricultural intensification and different farming practices may insert several contrasting ecological and economic impacts. From the ecological point of view, one of the fundamental characteristics of soil microbiomes is their capacity to withstand (resistance) and recover (resilience) from environmental stresses (Azarbad et al., 2016), which is often defined as “microbial stability” (see Philippot et al., 2021 for recent discussions on resistance, resilience, and stability concepts). Previous studies showed that soil microbial functional (process rates or functional genes) and/or community structural stabilities (species present in the community and their abundance) under rapidly changing climates might depend on the management histories of agricultural fields (de Vries et al., 2012; Fuchslueger et al., 2019; Piton et al., 2021). Furthermore, a recent survey summarizing the impacts of global environmental change (e.g., drought, temperature, nitrogen deposition, and salinity) on soil biota has shown that in general, soil ecologists have considered only one (80%) or two stress factors (19%) in their experiments (Rillig et al., 2019). This highlights the importance of considering multiple stress factors for better understanding the impact of global environmental change on soil biota. It is of ecological interest to determine which soil microbial groups are selected within the microbial community under selective pressure by different agricultural managements and climate conditions and how such selection shapes the resistance and resilience of the soil microbiome in the face of additional environmental stressors. However, the interactive effects of climate change-related stressors (e.g., drought and warming) and agricultural management on soil microbial stability in face of global change stressors are less studied and still poorly understood.

**FIGURE 2:**
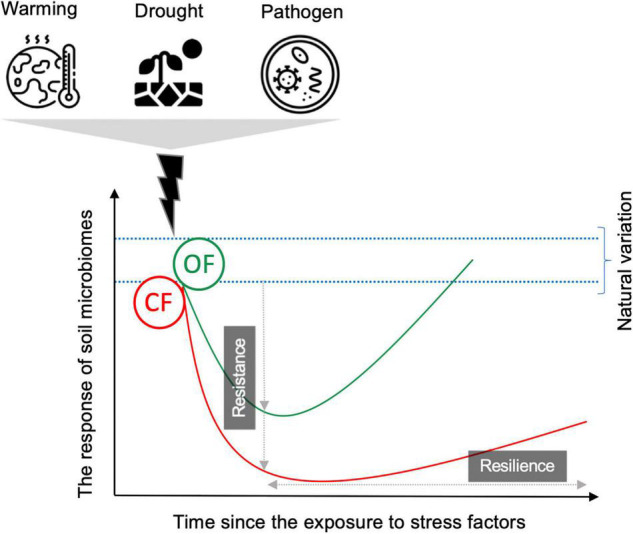
Conceptual figure showing soil microbial stability, in terms of resistance and resilience, in organic (OF) and conventional (CF) farming systems in response to stressors (e.g., warming, drought, pathogen). Resistance refers to the extent to which microbial communities withstand changes after exposure to stress factors (immediate or sudden responses). Resilience is the capacity of the microbiome to recover from the stress factor (the response over time). The response of the soil microbiome can be considered as changes in microbial function (process rates or functional genes) and/or community structure (species present in the community and their abundance). For details about possible scenarios on microbial stability, see the text.

Soil bacterial and fungi communities from agricultural fields with different management histories are expected to adapt to their local environmental conditions (Azarbad et al., 2018, 2020). Since different microbial groups have different degrees of stress tolerance, long-term soil management effects and exposure to a certain global change stressor may shift soil microbial profiles toward resistant taxa, which can tolerate the given stress factor (Bouskill et al., 2013; Evans and Wallenstein, 2014). For instance, microbial communities exposed to water stress go through district physiological mechanisms such as the accumulation of solutes together with polysaccharides and spores production (Schimel et al., 2007; Allison and Martiny, 2008), ensuring resistance to further water limitation (Azarbad et al., 2018). On the basis of this view, one outcome of exposure to agricultural intensities (e.g., high dose of chemical pesticides, N fertilizers, and frequent soil tillage) is a selection toward a microbial community with increasing tolerance to these stress factors. Such microbial selection may provide a general improvement of microbial stability not only to the initial stressor but also to stress factors of different nature as the results of community co-tolerance due to adaptation and physiological changes (Azarbad et al., 2015). However, on the other hand, such local adaptation may cause lower relative fitness toward a new stressor, resulting in a trade-off or cost of adaptation. In this case, microbial stability is likely to be severely reduced after exposure to a second stressor (that is a stress factor different from the initial stressor) if the initial stress eliminates certain microbial species and selects a resistant one (Azarbad et al., 2015). Therefore, soil memory or the legacy effect of agricultural management and climate conditions can be critical when estimating how the soil microbiome responds to a subsequent perturbation such as those related to global warming. This is particularly important for soil microbes under CF. A high application of chemical and synthetic fertilizers and pesticides results in reducing soil microbial diversity and community heterogeneity, as discussed in the previous section, which may further narrow down the resistance and resilience of soil microbiomes (Figure 2). From another hand, higher microbial diversity under OF may ensure their functional and compositional stability in face of the range of perturbations (Figure 2). However, in the agroecosystem, the direct and interactive effects of different management strategies and the rapid rate of change in climatic conditions on the stability of soil microbial communities in the face of secondary stressors are less well studied, and empirical evidence is limited. In a recent study using soil from grassland ecosystems across Switzerland, France, and Portugal, Piton et al. (2021) examined the effect of more than 20 years of history of conventional and organic management on the resistance and resilience of soil microbiome to four rain regimes (dry, wet, intermittent, and control) under laboratory condition. Their results showed that conventional management appeared to increase the resistance (that is the response after 263 days of exposure to rain regimes) of bacterial biomass (determined based on PLFAs) and extracellular enzyme activities (enzymes degrading C-, N-, or P-rich substrates) compared to ecological management. However, on the other hand, they showed that under organic management (no synthetic fertilizers and low N inputs), bacterial biomass and enzyme activities revealed a better recovery after the stress (that is the response after 89 days since the experiment was set to normal rain regime), which was not evident under conventional management. The authors pointed out that in agroecosystem management, a trade-off between resistance and recovery of microbial characters needs to be taken into account if the aim is to protect soil biological function to provide ecosystem services. Future studies on the stability of soil microbiomes should consider such a trade-off in agricultural systems.

## The Effect of Agricultural Management on Plant Microbiomes

As discussed above, different management approaches impact the diversity and composition of soil microbiomes and the range of vital functions they perform, but how these effects expand to the plant microbiomes under different agroecosystems has received less attention so far. It has been shown that the beneficial microbes associated with varying parts of the plant, including plant growth-promoting bacteria (PGPB), are considered as extended plant phenotypes which play critical roles in improving nutrient uptake, inhibiting pathogens, and protecting plants against stresses (Li et al., 2019). Therefore, while management-induced shifts in soil microbiomes result in several environmental outcomes (e.g., greenhouse gas emissions and mediating soil organic carbon), changes in soil and plant-associated microbiomes may directly affect agricultural productivity (Schmidt et al., 2019).

In response to the local environments, plants change the quantity and quality of belowground C inputs through rhizodeposition or root exudation, releasing a range of organic compounds to the surrounding root environment. Microbes in the rhizosphere (soil attached to the roots), in turn, deliver the necessary nutrients for plant growth and development *via* nutrient turnover from the organic matter (Richardson et al., 2009; Berendsen et al., 2012). Root exudates encompass a wide range of primary (e.g., amino and organic acids) and secondary (e.g., terpenoids, flavonoids, and phenolics) metabolites. Furthermore, since microbes consume root exudates as a carbon source, plants may change microbial community composition toward more beneficial microbes to ensure their nutritional needs (Wattenburger et al., 2019) and cope with stressful conditions such as drought (Marasco et al., 2012). However, CF practice neglects such complex synergetic interactions that occur between plants and their microbiome and relies heavily on external inputs such as inorganic nutrients to provide these essential elements for plants.

Hartmann et al. (2015) studied soil and winter wheat root-associated microbiomes in a field experiment consisting of conventional and organic managements with different tillage intensities (no-tillage, reduced-tillage, and intensive tillage). Their results showed that the diversity of bacteria and fungi was highest in OF with intensive tillage for soil and root communities. In addition, recent work by Longley et al. (2020) represents one of the first studies to determine the effect of more than 30 years of conventional, no-till, and organic management systems on the soil and soybean-associated microbiomes (roots, stems, and leaves) throughout the plant development stages. Surprisingly, their results indicate that OF leads to lower microbial Shannon diversity (both fungal and bacterial). They further reported that no-till management increased the abundance of *Bradyrhizobium* and *Glomeromycotina* in the root, known as plant beneficial organisms. Using six paired tomato farms in northern California with conventional and organic practices, Schmidt et al. (2019) showed that bacterial and fungal diversity was higher in the rhizospheres of organically managed plants than in conventional management. In addition, several plant-growth-promoting bacteria (e.g., *Pseudomonas*) appeared to have a higher relative abundance in organic management farms (Schmidt et al., 2019). In a recent comprehensive study, Ricono et al. (2022) investigated the long-term effect of organic and conventional farming on winter wheat root-associated bacterial and fungal communities across 40 agricultural fields. Their result revealed that in comparison with CF, OF enhanced Shannon diversity of the root microbiomes with an increased abundance of symbiotic fungi (e.g., *Glomeromycota*) and disease suppressive bacteria such as *Pseudomonadaceae*, *Burkholderiaceae*, and *Xanthomonadales*, *Gammaproteobacteria* (Ricono et al., 2022). However, similar to the soil microbiomes, since the effects of different agricultural practices and climate change factors on microbes linked with an important component of the plants have often been studied separately, it remains to be answered how the combination of such factors impacts plant-associated microbial communities and diversities and subsequently plant growth and agricultural sustainably.

Although not the focus of this study, it is important to highlight that apart from beneficial interaction, climate change can alter the plant-pathogen interactions and thus may accelerate the emergence of new pathogens. In their review paper, Velásquez et al. (2018) discuss the importance of taking into account factors that are linked with global climate changes (e.g., water availability and temperature) to fully understand the complex plant-pathogen interactions. The critical question is which management strategy (organic or conventional) would be more susceptible to plant pathogens under global climate changes?

## The Application of (Complex) Microbiome-Based Approaches Toward Productive Agriculture

A different group of microbes may have pathogenic or beneficial features. Shifting the soil and plant microbiome toward more mutualistic and beneficial microorganisms has the potential to have a great impact on plant health and growth (Quiza et al., 2015; Agoussar and Yergeau, 2021). Plant hosts and their associated microbes can quickly adapt to stressful environmental conditions mainly *via* the following mechanisms: (a) acquisition of new microbial partners from external sources, (b) facilitation/reduction of the microbes that already exist in the plant environment, and (c) horizontal transfer of genes from the external microbes to resident microbes. Through a single or combination of these mechanisms, plant-associated microbes may affect phenotypic traits and eventually fitness of the plant host. However, it is challenging to modify and manipulate the microbial communities in the plant environment to improve host phenotypes and, ultimately, productivity.

Inoculation of a single or a few beneficial microbial isolates into the soil or the plant parts have been applied quite extensively in the past to enhance plant response to unfavorable environmental conditions. The output of such studies was often unsuccessful due to the lack of survival of microbial isolates or high competition with microbes that already exist in the soil and plant environment (Agoussar and Yergeau, 2021; Wang and Song, 2022). Although such studies shed more light on how soil-plant-microbes interact, there is a need to move toward complex microbiomes rather than single isolate applications to improve agricultural productivity (Raaijmakers and Mazzola, 2016). In this context, there are several excellent examples of how the disease-suppressive capacity of microbes can be transferred from one to another soil environment by mixing a tiny proportion of resistant soil with a larger amount of susceptible soil (Mendes et al., 2011). I argue that more attention needs to be paid to the complex beneficial soil and plant microbiomes that evolved over a certain period of time *via* multiple layers of interactions that take place between soil-microbes-plants under adverse environmental conditions as a basis to enhance plant productivity. An interesting example is a study done by Yuan et al. (2018), where they examined how soil bacterial communities are influenced by plants when leaves of *Arabidopsis thaliana* were inoculated with a pathogen (*Pseudomonas syringae*) for five generations. They observed that as the result of soil-memory effects plants that were exposed to the pathogen can select beneficial soil microbes through changes in their root exudation profiles, thus exhibiting higher resistance to the pathogen than control plants (Yuan et al., 2018). In support of this, our team demonstrated that wheat plants had higher root biomass, an important plant trait under drought stress, when grown in soil with a long-term history of water stress (Azarbad et al., 2018). These results suggest that complex soil microbes previously subjected to water limitations could help plants cope better with subsequent water stresses.

Another critical point highlighting the importance of considering the legacy of previous exposure of microbes to stress factors is the “stress gradient hypothesis” (Bertness and Callaway, 1994). Based on this hypothesis, positive interactions between different members of microbial species should be expected to occur in the higher range under stressful environments than in optimum conditions where competitions gain more importance. This hypothesis has been proven in several recent studies (Piccardi et al., 2019; Palmer and Foster, 2022) but has not been tested for microbes under different agricultural management in the face of multiple stress factors. Therefore, one promising approach is to expose plants to complex soil microbes originating from areas with historical stress conditions. More positive facilitation and relatively less competition among microbial species in such harsh environments are expected, which would help plants select which microbes are “adopted” for better growth under different stress scenarios. We also need to be aware that such microbiome-based approaches may also insert a negative impact. For instance, introducing a complex microbial community in the soil and plant environment may shift the keystone microbes present in the host, which are essential for plant health and growth that can ultimately change rhizodeposition and, thus, the properties of the surrounding soil environment. Such factors and possible changes need to be considered in future research.

On top of that, a less explored avenue in microbial roles for plant adaptation is the process of horizontal transfer in such a way that traits can be passed on from not-associated host microbes to the resident microbes. Niehus et al. (2015) claimed that rather than microbial species, it’s mainly genes that occupy competitive niches through horizontal transfer. Their findings suggest that species are exchangeable as long as they gain the key genes required to adjust to a distinct niche. However, to date, studies of horizontal or environmental transfer of genetic material touched the theoretical parts primarily, leaving a large gap in our understanding of how the horizontal transfer of critical genes would help the plant to adapt to the rapidly shifting environments. It needs to be mentioned that incorporating such an approach in organic-based farming to boost yields requires changes in governmental policies since biotechnology innovations are not applicable in most cases.

## Conclusion and Future Directions

**FIGURE 3:**
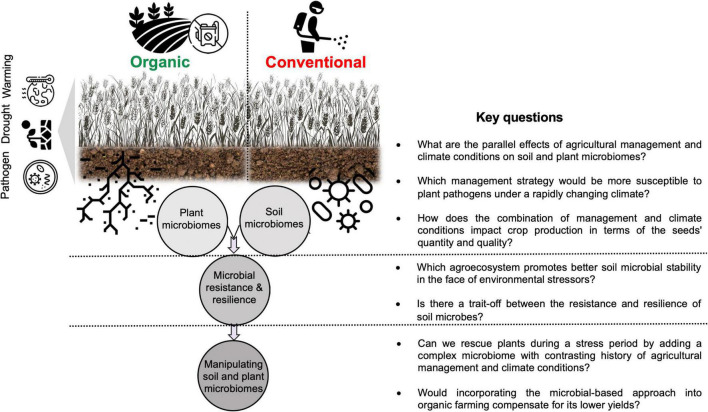
Key research questions and directions for future studies regarding the combined effects of agricultural management and multiple stressors on soil and plant microbiomes.

This article emphasizes the importance of considering soil-microbe-plant interactions under different agricultural management not only to a single stress factor (e.g., pathogen) but also to multiple stress factors (e.g., drought, warming, and salinity) for a better understanding of cumulative interactions between such factors and the consequent impact on crop yields. Data from limited literature in this important area of research indicate that the resistance and resilience response of soil microbial communities to climate change-related stressors depend on the management histories of agricultural fields. Under current and future global environmental changes, where temperature and water availability fluctuate very rapidly, it sounds plausible that higher microbial diversity and the presence of certain beneficial microbes in the soil environment under organic farming would ensure better soil resilience (e.g., in terms of important soil functions). Apart from the biotic factors, abiotic factors such as higher organic matter and the water capacity of soil managed organically, contribute greatly to better soil resilience. Future microbiome-based studies should consider such interactions between long-term exposure of soil microbial communities to the combination of several stress factors linked with climate change and different agricultural management strategies (Figure 3). In addition, it remains to be answered how a combination of such factors impacts not only the quantity of agricultural production (yields) but also the quality of the production (e.g., seeds quality such as seed size its protein levels). I propose to address these gaps by asking several critical questions which are listed in Figure 3.

I expect that addressing these questions will provide a better insight into whether incorporating the microbial-based approach into organic farming would compensate for its lower yields (in comparison with conventional farming), under global warming. If yes, then it will ensure the advantage of organic farming over conventional (in terms of yields, in particular) without massive land-use changes and expansion of farmland. Since agricultural intensification, land-use changes and ongoing climate changes are important, and yet unsolved global issues, this line of research is critical to avoid further damage to natural ecosystems and, at the same time, maintain or enhance agricultural productivity. This would not be possible without the active engagement of the public and bringing together the knowledge of various disciplines such as plant breeding, microbiologist, decision-makers, and many other relevant fields.

## Author Contributions

The author confirms being the sole contributor of this work and has approved it for publication.

## Conflict of Interest

The author declares that the research was conducted in the absence of any commercial or financial relationships that could be construed as a potential conflict of interest.

## Publisher’s Note

All claims expressed in this article are solely those of the authors and do not necessarily represent those of their affiliated organizations, or those of the publisher, the editors and the reviewers. Any product that may be evaluated in this article, or claim that may be made by its manufacturer, is not guaranteed or endorsed by the publisher.
